# Prealbumin as a Predictor of Prognosis in Patients With Coronavirus Disease 2019

**DOI:** 10.3389/fmed.2020.00374

**Published:** 2020-06-26

**Authors:** Ying Luo, Ying Xue, Liyan Mao, Xu Yuan, Qun Lin, Guoxing Tang, Huijuan Song, Feng Wang, Ziyong Sun

**Affiliations:** ^1^Department of Laboratory Medicine, Tongji Hospital, Tongji Medical College, Huazhong University of Science and Technology, Wuhan, China; ^2^Department of Clinical Immunology, Tongji Hospital, Tongji Medical College, Huazhong University of Sciences and Technology, Wuhan, China

**Keywords:** coronavirus disease 2019, severe acute respiratory syndrome coronavirus 2, prealbumin, routine laboratory tests, prognosis, immune status, fatal patients, recovered patients

## Abstract

**Background:** The predictive value of prealbumin for the prognosis of coronavirus disease 2019 (COVID-19) has not been extensively investigated.

**Methods:** A total of 1,115 patients with laboratory-confirmed COVID-19 were enrolled at Tongji hospital from February to April 2020 and classified into fatal (*n* = 129) and recovered (*n* = 986) groups according to the patient's outcome. Prealbumin and other routine laboratory indicators were measured simultaneously.

**Results:** The level of prealbumin on admission was significantly lower in fatal patients than in recovered patients. For predicting the prognosis of COVID-19, the performance of prealbumin was better than most routine laboratory indicators, such as albumin, lymphocyte count, neutrophil count, hypersensitive C-reactive protein, d-dimer, lactate dehydrogenase, creatinine, and hypersensitive cardiac troponin I. When a threshold of 126 mg/L was used to discriminate between fatal and recovered patients, the sensitivity and specificity of prealbumin were, respectively, 78.29 and 90.06%. Furthermore, a model based on the combination of nine indexes showed an improved performance in predicting the death of patients with COVID-19. Using a cut-off value of 0.19, the prediction model was able to distinguish between fatal and recovered individuals with a sensitivity of 86.82% and a specificity of 90.37%.

**Conclusions:** A lower level of prealbumin on admission may indicate a worse outcome of COVID-19. Immune and nutritional status may be vital factors for predicting disease progression in the early stage of COVID-19.

## Introduction

Coronavirus disease 2019 (COVID-19) as a rampant disease caused by the emerging infection of severe acute respiratory syndrome coronavirus 2 (SARS-CoV-2), the outbreak of which has initiated an extreme health concern (1, 2). Many patients might progress to acute respiratory disease or other complications in a short period of time (3, 4). Since no effective vaccine or anti-viral treatment is currently available, this epidemic is difficult to manage and control. As of 7 May 2020, the number of patients infected with SARS-CoV-2 has exceeded 3.5 million globally, and there has been more than 250,000 reported cases of COVID-19-related deaths worldwide (5). Therefore, it is urgent and desirable to establish a model which can be used to predict the progression of disease and help clinicians to better choose a therapeutic strategy.

Up to now, many studies have reported that the risk factors for death in patients with COVID-19 are attributed to advanced age and co-morbidities including hypertension, myocardial injury, liver damage, and kidney failure (6–10). Some indicators such as creatinine (CR), lactate dehydrogenase (LDH), aspartate aminotransferase (AST), and hypersensitive cardiac troponin I (hs-cTnI) have been found to be helpful to assess the severity of the disease and predict the prognosis of COVID-19 (9, 11, 12). In addition, some studies discovered and elaborated the potential value of coagulation indicators represented by prothrombin time (PT) and d-dimer (DD) in predicting the prognosis and outcome of patients with COVID-19 (13–15). Moreover, some recent studies focused on the body's immune status, including inflammatory responses and the number and phenotype of lymphocytes, and found that some inflammatory indicators or cytokines including hypersensitive C-reactive protein (hsCRP), procalcitonin (PCT), interleukin-6, interleukin-2 receptor, interferon-gamma, and the number of lymphocytes also contributed to the outcome of the disease (16–19).

Additionally, some indicators, such as albumin (ALB) and prealbumin (PAB), can partially reflect the nutritional and immune status of the host (20–23). However, the potential value of them for the prognosis of COVID-19 has not been fully explored. Theoretically, in view of the decrease in the number of lymphocytes and their subsets in the early stage of the disease (24, 25) and the poor immune function potentially caused by various complications (26, 27), ALB and PAB are potential and feasible predictors for the prognosis of COVID-19. In this study, we did a comprehensive evaluation of various laboratory indicators in fatal and recovered patients with confirmed COVID-19 on admission. We also compared the predictive value of PAB and other routine laboratory makers for the prognosis of COVID-19. It is hoped that the information obtained in this study will offer a better understanding on the disease progression that occurs after SARS-CoV-2 infection, and establish a basis to optimize the current treatment.

## Methods

### Study Design and Participants

The current study was conducted at Tongji hospital (the largest hospital in the central region of China) in Wuhan, China. Consecutive hospitalized patients with confirmed COVID-19 were enrolled between February and April 2020. COVID-19 was diagnosed if patients met the following criteria: (1) having typical clinical symptoms, (2) having typical imaging findings, and (3) positive for SARS-CoV-2 real-time reverse transcription-polymerase chain reaction. The patients who died during hospitalization were defined as the fatal group, and those who recovered and were finally discharged after hospitalization were defined as the recovered group. All recovered patients with COVID-19 met the following criteria: having completely resolved symptoms and signs, having significant improvement in pulmonary and extrapulmonary organ dysfunction, no longer need supportive care, and with confirmed viral clearance by repeated tests for SARS-CoV-2 before hospital discharge. This study was reviewed and approved by the ethical committee of Tongji Hospital, Tongji Medical College, Huazhong University of Science and Technology, Wuhan, China (TJ-C20200128).

### Real Time Reverse Transcription-Polymerase Chain Reaction

The clinical samples, including throat and nasal swab obtained from patients at admission or during the hospital stay, were maintained in a viral-transport medium. SARS-CoV-2 was confirmed by using TaqMan One-Step reverse transcription-polymerase chain reaction (RT-PCR) Kits from Shanghai Huirui Biotechnology Co., Ltd and Shanghai BioGerm Medical Biotechnology Co., Ltd. Briefly, RNA was extracted from clinical samples. 5 μL of RNA was used for real-time RT-PCR, which targeted the ORF1ab and N gene. Real-time RT-PCR was performed using the following conditions: 50°C for 15 min and 95°C for 5 min, 45 cycles of amplification at 95°C for 10 s and 55°C for 45 s. The positive SARS-CoV-2 real time RT-PCR result was defined if both ORF1ab and N cycle thresholds were <35.

### Laboratory Tests

The measurements of white blood cell count (WBC#), neutrophil count (NEU#), lymphocyte count (LYM#), and platelet count (PLT#) were performed using XN-9000 Sysmex (Sysmex Co., Kobe, Japan). The measurements of total protein (TP), PAB, ALB, globulin (GLB), CR, AST, LDH, hsCRP, PCT, and hs-cTnI were performed using ROCHE COBAS 8000 (Mannheim, Germany). PT and DD were detected using STA-R coagulation analyzers (Diagnostic Stago, France) according to the manufacturer's instructions.

### Statistical Analysis

Continuous variables were expressed as mean ± standard deviation (SD) and categorical variables were reported as numbers and percentages (%). The comparison between continuous variables was performed using the Wilcoxon test or Mann-Whitney *U* test. The chi-square test was used for comparison of categorical data. The area under the curves (AUCs) were compared using the z statistic with the procedure of (28). A two-sided *P*-value below 0.05 was considered to be statistically significant. A prediction model for predicting the outcome of death was established by using a multivariate logistic regression method. All variables with statistical significance were taken as candidates for multivariable logistic regression analyses, and the regression equation (prediction model) was obtained. The regression coefficients of the prediction model were regarded as the weights for the respective variables and a score for each patient was calculated. Receiver operating characteristic (ROC) analysis was performed on these scores to assess the ability for distinguishing between fatal and recovered COVID-19 patients. AUC, sensitivity, specificity, positive predictive value (PPV), negative predictive value (NPV), positive likelihood ratio (PLR), and negative likelihood ratio (NLR), together with their 95% confidence intervals (CI), were calculated. Data were analyzed by using SPSS version 25.0 (SPSS, Chicago, IL, USA), GraphPad Prism version 6 (GraphPad Software, San Diego, CA, USA), and MedCalc version 11.6 (Medcalc, Mariakerke, Belgium).

## Results

### Demographic and Clinical Characteristics of Study Participants

Our study enrolled 129 patients who died during hospitalization and 986 recovered patients (Table 1). There were 552 women (49.51%) and 563 men (50.49%) in this cohort, with ages ranging from 16 to 95 years old. The mean age of fatal patients (69.98 ± 12.05 years) was significantly older than recovered patients (58.64 ± 15.17 years) (*P* < 0.001). Male sex was more predominant in fatal patients (67.44%) than in recovered patients (48.28%) (*P* < 0.001). Cough and fever were the most prevalent symptoms at disease onset in both fatal (51.16 and 62.02%) and recovered patients (58.42 and 65.72%). Other prevalent symptoms at the onset of illness in fatal patients included shortness of breath and chest tightness; less common symptoms included diarrhea, headache, nausea, vomiting, muscle ache, and pharyngalgia. Shortness of breath was significantly higher in fatal patients (34.88%) than in recovered patients (12.78%) (*P* < 0.001). Underlying diseases including diabetes mellitus, hypertension, chronic obstructive pulmonary disease, cardiovascular disease, and hematological malignancy were more frequent in fatal patients (19.38, 43.41, 3.88, 17.83, and 1.15%) than in recovered patients (7.3, 25.56, 1.42, 9.63, and 0.2%). The mean time from admission to death was 16.72 ± 11.9 days in fatal patients. The mean time from admission to discharge was 21.66 ± 12.01 days in recovered patients (Table 1).

**Table 1:** Demographic and clinical characteristics of study participants.

**Variables**	**Fatal (*n* = 129)**	**Recovered (*n* = 986)**	P^*^
Sex, male, %	87 (67.44%)	476 (48.28%)	<0.001
**Age, years**			
Mean ± SD	69.98 ± 12.05	58.64 ± 15.17	<0.001
<50	4 (3.10%)	251 (25.46%)	<0.001
50–59	16 (12.40%)	208 (21.1%)	0.021
60–69	40 (31.01%)	300 (30.43%)	0.893
70–79	40 (31.01%)	149 (15.11%)	<0.001
>79	29 (22.48%)	78 (7.91%)	<0.001
**Symptoms on admission**			
Cough	66 (51.16%)	576 (58.42%)	0.117
Fever	80 (62.02%)	648 (65.72%)	0.406
Shortness of breath	45 (34.88%)	126 (12.78%)	<0.001
Chest tightness	25 (19.38%)	198 (20.08%)	0.852
Diarrhea	8 (6.20%)	108 (10.95%)	0.096
Headache	2 (1.55%)	27 (2.74%)	0.425
Nausea and vomiting	7 (5.43%)	36 (3.65%)	0.325
Muscle ache	7 (5.43%)	54 (5.48%)	0.981
Pharyngalgia	3 (2.33%)	45 (4.56%)	0.24
**Underlying condition or illness**			
Diabetes mellitus	25 (19.38%)	72 (7.30%)	<0.001
Hypertension	56 (43.41%)	252 (25.56%)	<0.001
Chronic obstructive pulmonary disease	5 (3.88%)	14 (1.42%)	0.043
Cardiovascular disease	23 (17.83%)	95 (9.63%)	0.004
Chronic kidney disease	6 (4.65%)	32 (3.25%)	0.408
Chronic liver disease	3 (2.33%)	41 (4.16%)	0.315
Hematological malignancy	2 (1.55%)	2 (0.20%)	0.016
Solid tumor	6 (4.65%)	50 (5.07%)	0.837
Organ transplantation	1 (0.78%)	5 (0.51%)	0.696
**Days from admission to death**			
Mean ± SD	16.72 ± 11.90	N/A	N/A
<3	7 (5.43%)	N/A	N/A
3–7	20 (15.50%)	N/A	N/A
8–14	43 (33.33%)	N/A	N/A
15–30	41 (31.78%)	N/A	N/A
>30	18 (13.95%)	N/A	N/A
**Days from admission to discharge**			
Mean ± SD	N/A	21.66 ± 12.01	N/A
<3	N/A	3 (0.30%)	N/A
3–7	N/A	98 (9.94%)	N/A
8–14	N/A	241 (24.44%)	N/A
15–30	N/A	404 (40.97%)	N/A
> 30	N/A	240 (24.34%)	N/A

*N/A, not applicable*.

* *Comparisons were performed between fatal and recovered groups using chi-square test or Mann-Whitney U test. Data were presented as means ± SD or numbers (percentages)*.

### Using PAB for Predicting the Prognosis of COVID-19

**Figure 1:**
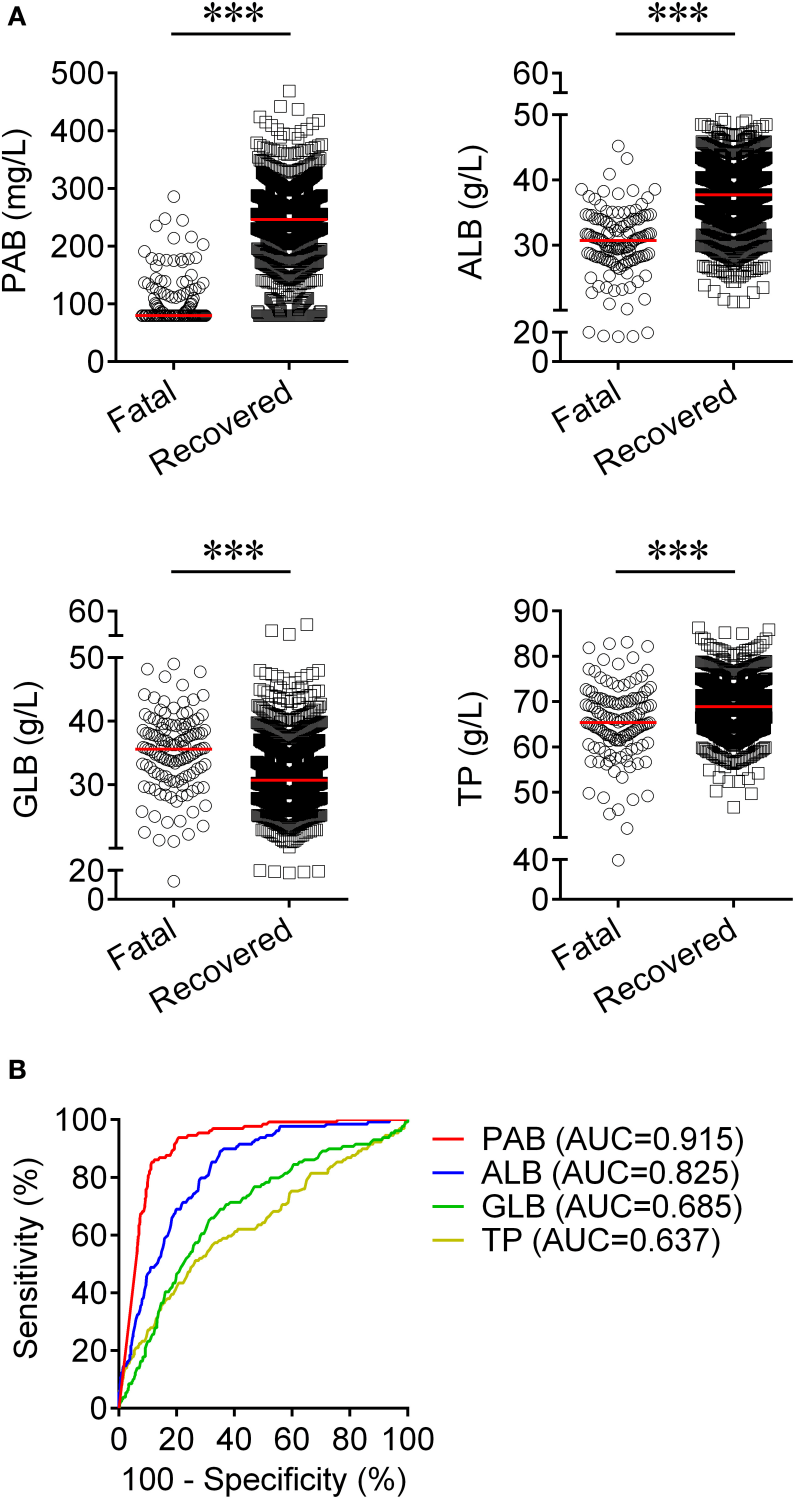
Using PAB on admission for discriminating fatal patients from recovered patients. **(A)** Scatter plots showing the concentrations of PAB, ALB, GLB, and TP in fatal (*n* = 129) and recovered (*n* = 986) patients. Horizontal lines indicate the median. ^***^P < 0.001 (Mann-Whitney *U* test). **(B)** ROC analysis showing the performance of PAB, ALB, GLB, and TP in distinguishing fatal patients from recovered patients. PAB, prealbumin; ALB, albumin; GLB, globulin; TP, total protein; ROC, receiver operating characteristic curve; AUC, area under the curve.

We observed substantial differences in the levels of proteins including PAB, ALB, GLB, and TP between patients who died of COVID-19 and those who recovered from the disease. It was found that the concentrations of PAB, ALB, and TP on admission were markedly lower in fatal patients than in recovered patients (*P* < 0.001). On the contrary, the level of GLB on admission was found to be significantly higher in the fatal group than in the recovered group (*P* < 0.001) (Figure 1A). If using these indexes for distinguishing these two conditions, the best AUC was for PAB [0.915, (95% CI, 0.894–0.937)] (Table 2, Figure 1B). Notably, the level of PAB ≤ 126 mg/L produced a sensitivity of 78.29 % and a specificity of 90.06% (Table 2). In addition, ROC analysis showed that the AUC of ALB was 0.825 (95% CI, 0.792–0.859), with a sensitivity of 45.74% and a specificity of 90.37% when a cutoff value of 29.7 g/L was used to differentiate between fatal and recovered individuals (Table 2, Figure 1B).

**Table 2:** The performance of various methods for distinguishing between fatal and recovered patients.

**Variables**	**Cutoff value**	**AUC (95% CI)**	**Sensitivity (95% CI)**	**Specificity (95% CI)**	**PPV (95% CI)**	**NPV (95% CI)**	**PLR (95% CI)**	**NLR (95% CI)**
PAB (mg/L)	126	0.915 (0.894–0.937)	78.29% (71.18%−85.41%)	90.06% (88.19%−91.93%)	50.75% (43.81%−57.70%)	96.94% (95.83%−98.06%)	7.88 (6.39–9.71)	0.24 (0.17–0.33)
ALB (g/L)	29.7	0.825^†^ (0.792–0.859)	45.74% (37.14%−54.33%)	90.37% (88.52%−92.21%)	38.31% (30.63%−45.99%)	92.72% (91.07%−94.36%)	4.75 (3.63–6.21)	0.60 (0.51–0.70)
GLB (g/L)	39.4	0.685^†^ (0.636–0.735)	22.48% (15.28%−29.68%)	90.26% (88.41%−92.11%)	23.20% (15.80%−30.60%)	89.90% (88.02%−91.78%)	2.31 (1.59–3.35)	0.86 (0.78–0.94)
TP (g/L)	61.4	0.637^†^ (0.582–0.693)	24.81% (17.35%−32.26%)	90.47% (88.63%−92.30%)	25.40% (17.80%−33.00%)	90.19% (88.34%−92.05%)	2.60 (1.82–3.72)	0.83 (0.75–0.92)
WBC# (×10^9^/L)	9.83	0.751^†^ (0.698–0.804)	45.74% (37.14%−54.33%)	90.77% (88.96%−92.58%)	39.33% (31.52%−47.15%)	92.75% (91.11%−94.38%)	4.96 (3.78–6.50)	0.60 (0.51–0.70)
LYM# (×10^9^/L)	0.6	0.842^†^ (0.807–0.877)	46.51% (37.90%−55.12%)	90.06% (88.19%−91.93%)	37.97% (30.41%−45.54%)	92.79% (91.15%−94.43%)	4.68 (3.59–6.09)	0.59 (0.50–0.70)
NEU# (×10^9^/L)	7.9	0.805^†^ (0.757–0.853)	48.84% (40.21%−57.46%)	91.18% (89.41%−92.95%)	42.00% (34.10%−49.90%)	93.16% (91.57%−94.75%)	5.53 (4.24–7.23)	0.56 (0.47–0.66)
PLT# (×10^9^/L)	145	0.722^†^ (0.667–0.776)	44.19% (35.62%−52.76%)	90.16% (88.30%−92.02%)	37.01% (29.39%−44.64%)	92.51% (90.84%−94.17%)	4.49 (3.43–5.89)	0.62 (0.53–0.72)
PCT (ng/mL)	0.22	0.898^¶^ (0.873–0.923)	58.91% (50.42%−67.40%)	90.16% (88.30%−92.02%)	43.93% (36.53%−51.33%)	94.37% (92.90%−95.85%)	5.99 (4.72–7.60)	0.46 (0.37–0.56)
hsCRP (mg/L)	89	0.880^§^ (0.853–0.908)	58.14% (49.63%−66.65%)	90.06% (88.19%−91.93%)	43.35% (35.97%−50.74%)	94.27% (92.78%−95.75%)	5.85 (4.61–7.42)	0.46 (0.38–0.57)
PT (s)	15.1	0.839^†^ (0.798–0.879)	58.14% (49.63%−66.65%)	91.48% (89.74%−93.22%)	47.17% (39.41%−54.93%)	94.35% (92.89%−95.81%)	6.82 (5.31–8.78)	0.46 (0.37–0.56)
DD (mg/L)	3.9	0.866^‡^ (0.837–0.896)	55.81% (47.24%−64.38%)	90.57% (88.74%−92.39%)	43.64% (36.07%−51.20%)	94.00% (92.49%−95.51%)	5.92 (4.62–7.58)	0.49 (0.40–0.59)
LDH (U/L)	428	0.866^‡^ (0.830–0.902)	59.69% (51.23%−68.15%)	91.99% (90.29%−93.68%)	49.36% (41.51%−57.20%)	94.58% (93.14%−96.01%)	7.45 (5.78–9.61)	0.44 (0.35–0.54)
AST (U/L)	51	0.753^†^ (0.709–0.797)	34.88% (26.66%−43.11%)	90.06% (88.19%−91.93%)	31.47% (23.86%−39.08%)	91.36% (89.59%−93.12%)	3.51 (2.60–4.74)	0.72 (0.64–0.82)
CR (μmol/L)	100	0.711^†^ (0.659–0.764)	37.98% (29.61%−46.36%)	90.06% (88.19%−91.93%)	33.33% (25.71%−40.95%)	91.74% (90.00%−93.47%)	3.82 (2.86–5.11)	0.69 (0.60–0.79)
hs–cTnI (pg/mL)	30.1	0.864^‡^ (0.831–0.898)	58.91% (50.42%−67.40%)	90.97% (89.18%−92.76%)	46.06% (38.46%−53.67%)	94.42% (92.96%−95.88%)	6.53 (5.11–8.34)	0.45 (0.37–0.56)
Prediction model	0.19	0.955 (0.941–0.970)	86.82% (80.98%−92.66%)	90.37% (88.52%−92.21%)	54.11% (47.32%−60.89%)	98.13% (97.25%−99.01%)	9.01 (7.36–11.04)	0.15 (0.09–0.23)

† *P < 0.001, compared with PAB using the z statistic*;

‡ *P < 0.01, compared with PAB using the z statistic*;

§ *P < 0.05, compared with PAB using the z statistic*;

¶ *P > 0.05, compared with PAB using the z statistic*;

*AUC, area under the curve; PPV, positive predictive value; NPV, negative predictive value; PLR, positive likelihood ratio; NLR, negative likelihood ratio; CI, confidence interval; PAB, prealbumin; ALB, albumin; GLB, globulin; TP, total protein; WBC#, white blood cell count; LYM#, lymphocyte count; NEU#, neutrophil count; PLT#, platelet count; PCT, procalcitonin; hsCRP, hypersensitive C-reactive protein; PT, prothrombin time; DD, d-dimer; LDH, lactate dehydrogenase; AST, aspartate aminotransferase; CR, creatinine; hs-cTnI, hypersensitive cardiac troponin I*.

### Change of the Level of PAB in the Same Patients

**Figure 2:**
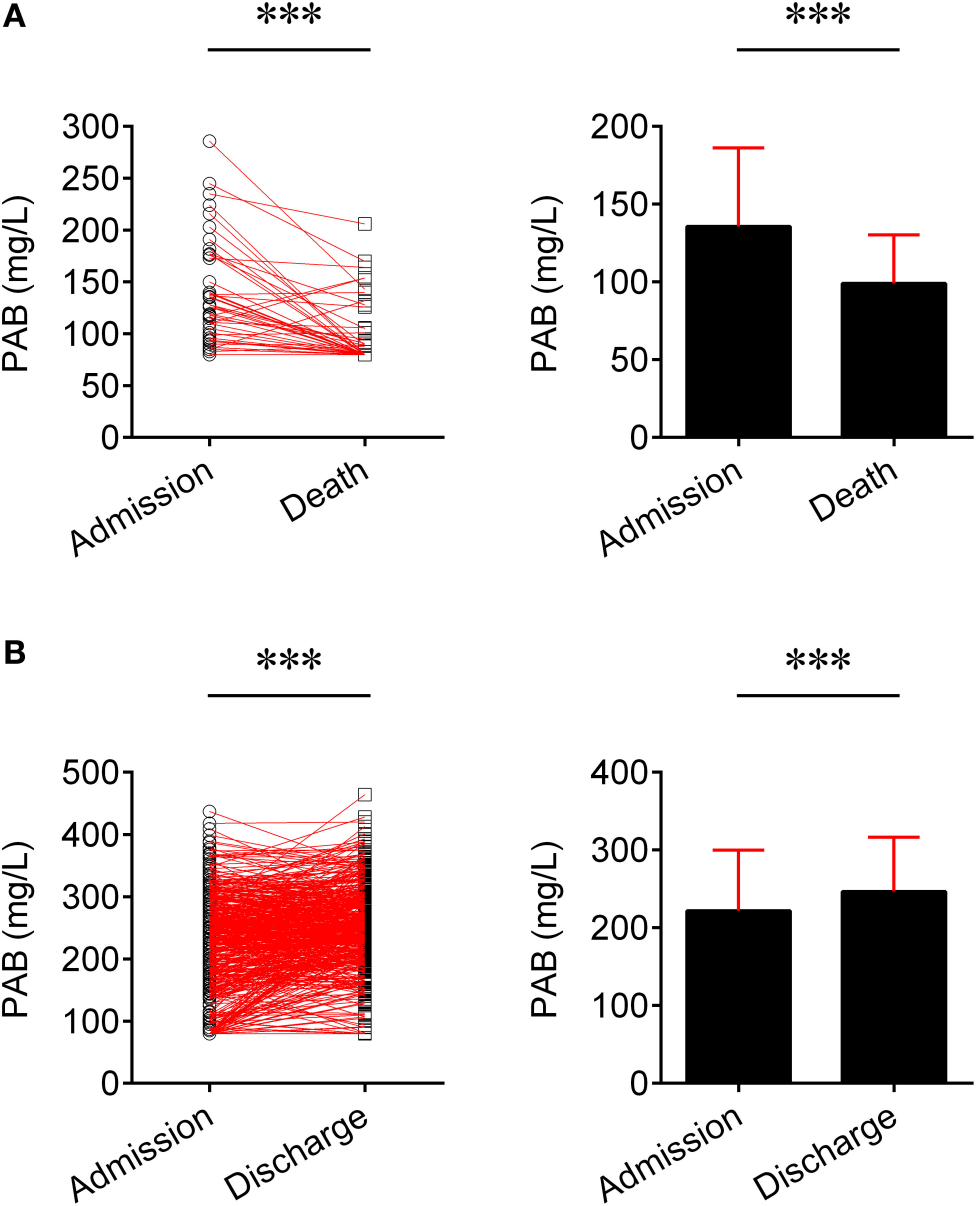
Change of the level of PAB in the same patients. **(A)** Line graphs showing the level of PAB for each fatal patient on admission and death (*n* = 45). One line represents one patient. ^***^P < 0.001 (Wilcoxon test). Bar graphs showing the level of PAB on admission and death in fatal patients (*n* = 45). Data are shown as means ± SD. ^***^P < 0.001 (Wilcoxon test). **(B)** Line graphs showing the level of PAB for each recovered patient on admission and discharge (*n* = 501). One line represents one patient. ^***^P < 0.001 (Wilcoxon test). Bar graphs showing the level of PAB on admission and discharge in recovered patients (*n* = 501). Data are shown as means ± SD. ^***^P < 0.001 (Wilcoxon test). PAB, prealbumin.

We compared the level of PAB in fatal patients between admission and death. It was found that the level of PAB was significantly decreased at the time of death compared to admission (*P* < 0.001) (Figure 2A). Furthermore, the concentration of PAB was further compared between admission and discharge in recovered individuals. Conversely, recovered patients showed a significantly higher level of PAB on discharge compared with on admission (*P* < 0.001) (Figure 2B).

### The Comparison of Predictive Value Between PAB and Other Routine Laboratory Indicators for the Prognosis of COVID-19

**Figure 3:**
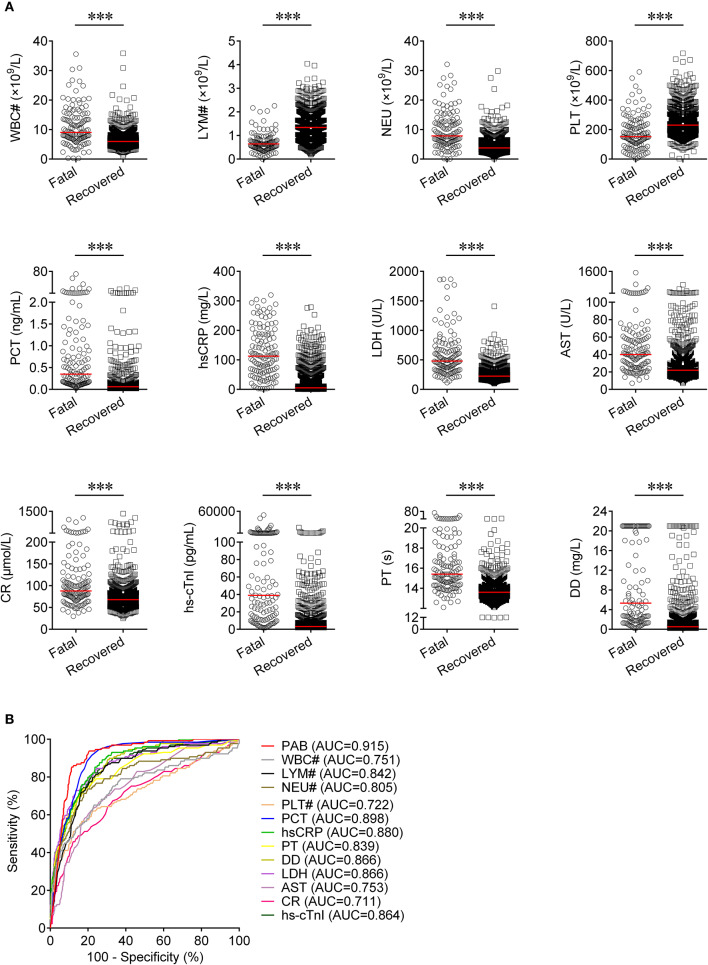
The comparison of predictive values between PAB and other route laboratory markers for the prognosis of COVID-19. **(A)** Scatter plots showing the levels of WBC#, LYM#, NEU#, PLT#, PCT, hsCRP, PT, DD, LDH, AST, CR, and hs-cTnI in fatal (*n* = 129) and recovered (*n* = 986) patients. Horizontal lines indicate the median. ^***^P < 0.001 (Mann-Whitney *U* test). **(B)** ROC analysis showing the performance of PAB, WBC#, LYM#, NEU#, PLT#, PCT, hsCRP, PT, DD, LDH, AST, CR, and hs-cTnI in distinguishing fatal patients from recovered patients. COVID-19, coronavirus disease 2019; PAB, prealbumin; WBC#, white blood cell count; LYM#, lymphocyte count; NEU#, neutrophil count; PLT#, platelet count; PCT, procalcitonin; hsCRP, hypersensitive C-reactive protein; PT, prothrombin time; DD, d-dimer; LDH, lactate dehydrogenase; AST, aspartate aminotransferase; CR, creatinine; hs-cTnI, hypersensitive cardiac troponin I; ROC, receiver operating characteristic curve; AUC, area under the curve.

Routine laboratory markers including WBC#, LYM#, NEU#, PLT#, PCT, hsCRP, PT, DD, LDH, AST, CR, and hs-cTnI were also measured in both fatal and recovered patients on admission. LYM# and PLT# were significantly lower in the fatal group than in the recovered group (*P* < 0.001) (Figure 3A). Conversely, it was found that WBC#, NEU#, PCT, hsCRP, PT, DD, LDH, AST, CR, and hs-cTnI in the fatal group was significantly higher than in the recovered group (*P* < 0.001) (Figure 3A). ROC analysis showed that the AUCs of LYM#, NEU#, PCT, hsCRP, PT, DD, LDH, and hs-cTnI were over 0.8 for distinguishing between fatal patients and recovered subjects (Figure 3B). Using a cut-off value of 0.22 ng/ml, the sensitivity and specificity of PCT for discriminating fatal cases from recovered individuals were 58.91 and 90.16%, respectively (Table 2). With a threshold of 89 mg/L, hsCRP was able to distinguish fatal patients from recovered patients with a sensitivity of 58.14% and a specificity of 90.06% (Table 2). Moreover, with a threshold of 15.1 s, PT had an AUC of 0.839 (95% CI, 0.798–0.879) with a sensitivity of 58.14% and a specificity of 91.48% (Table 2). The predictive utility of PAB was better than WBC#, LYM#, NEU#, PLT#, hsCRP, PT, DD, LDH, AST, CR, and hs-cTnI, and was comparable to PCT for the prognosis of patients with COVID-19 (Table 2).

### Establishing a Model for Predicting the Death of Patients With COVID-19

**Figure 4:**
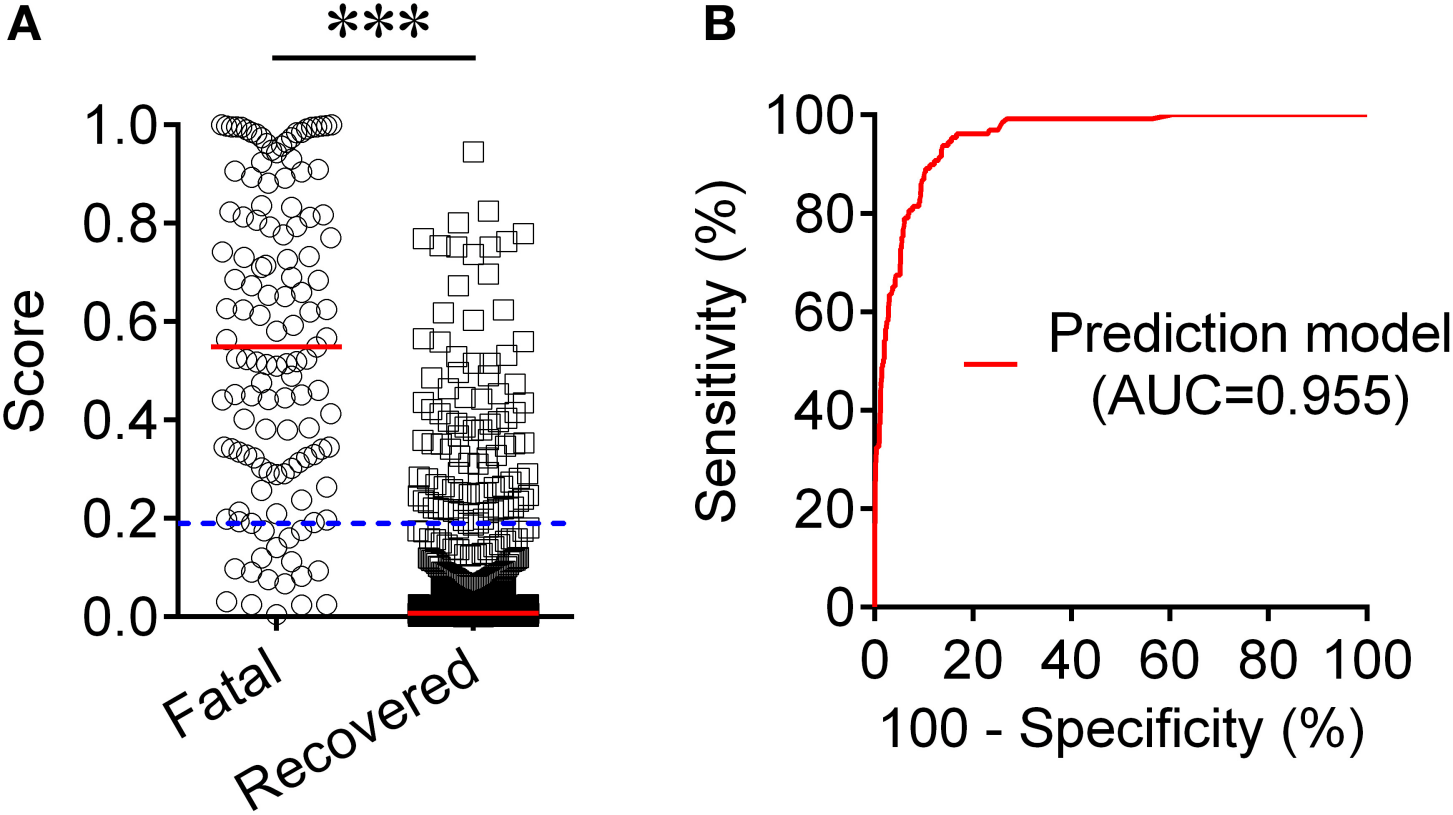
Establishment of prediction model for prognosis of COVID-19 based on the combination of PAB and other routine laboratory indexes. **(A)** Scatter plots showing the score of prediction model in fatal patients (*n* = 129) and survived cases (*n* = 986). Horizontal lines indicate the median. ^***^P < 0.001 (Mann-Whitney *U* test). Blue dotted lines indicate the cutoff value in distinguishing these two groups. **(B)** ROC analysis showing the performance of the prediction model in distinguishing fatal patients from recovered cases. COVID-19, coronavirus disease 2019; ROC, receiver operating characteristic curve; AUC, area under the curve.

To establish a prediction model based on the combination of PAB and other routine laboratory markers on admission for distinguishing fatal patients from recovered individuals, all variables with statistical significance were used for multivariable logistic regression analysis. A prediction model was built as the following: *P* = 1/[1 + e^−(−0.016*PAB−0.908*LYM#+0.067*NEU#+0.06*PCT+0.005*hsCRP+0.154*PT +0.003*LDH+0.002*CR+0.001*hs−cTnI−3.036)^] P, predictive value; e, natural logarithm (Supplementary Table 1). ROC analysis showed that the AUC of the prediction model was 0.955 (95% CI, 0.941-0.970) (Figure 4). When the cutoff value was set at 0.19, the following diagnostic parameters of the model were obtained: sensitivity, 86.82% (95% CI, 80.98–92.66%); specificity, 90.37% (95% CI, 88.52–92.21%) (Table 2). These data suggested that our established model, based on the combination of a nine-indicator biosignature, had good performance for predicting the death of patients with COVID-19.

## Discussion

The rapidly increasing number of laboratory-confirmed COVID-19 cases worldwide has put a heavy burden on the medical resources in countries with large outbreaks (29–31). The World Health Organization has declared COVID-19 to be a public health emergency of international concern. The determination of the outcome of the disease is of crucial importance in regulating limited medical resources and providing better care for patients (32, 33). Meanwhile, progression at the early stage is very important to the outcome or the prognosis of the disease. Therefore, after patient admission, identifying predictors that can predict the likelihood of disease progression would help physicians to decide which group of patients can be managed safely at district hospitals and who needs early transfer to tertiary centers.

Although previous studies have found that a series of parameters on admission are correlated with mortality risk (34), there is limited information in the existing literature regarding the relationship between nutrition indexes and disease progression in SARS-CoV-2-infected patients. Several studies have reported the value of nutritional indicators represented by PAB on determining the severity of viral infections and predicting the prognosis of patients (35, 36). In addition, there is some literature describing the roles of inflammatory indicators (hsCRP and PCT), coagulation indicators (DD), and the number of lymphocytes in monitoring the disease progression during viral infection and prediction of disease outcome (36–38). Our study comprehensively described the differences in PAB and other routine laboratory parameters between the patients who died of COVID-19 and those who recovered from the disease. It was found that a low level of PAB on admission may indicate a poor prognosis and that the predictive value of PAB is superior to most routine laboratory indicators that reflect functional impairment or disorder of organs for prognosis of COVID-19. To our knowledge, this is the first study to report the relationship between PAB level and the outcome of COVID-19.

Host factors trigger an immune response against the pathogens during viral infection (39–41). Immune insufficiency may contribute to viral replication and cause tissue damage, resulting in a bad outcome (42, 43). The systematic overwhelming inflammation and multi-organ dysfunction are more common in deceased COVID-19 patients than in recovered patients (44). This may be caused by the poor basic immune status of patients. In accordance with recent reports, advanced age and comorbidities such as diabetes and hypertension are believed to be risk factors of death from COVID-19 (45). This suggests that immune and nutritional status may be critical in predicting disease progression at the early stage of COVID-19. In other words, poor immunity may play a role in COVID-19-related death. Thus, early vigilant monitoring along with high quality supportive care are needed for patients at high risk of death. Although the number of lymphocytes can partially reflect the host's immune function, our data indicates that the nutritional indicators represented by PAB may show this effect better. A single use of PAB could achieve a modest prediction performance for the prognosis of COVID-19. When combined with other conventional laboratory indicators, PAB could produce a better performance.

In addition, opposite trends were found in PAB level between fatal and recovered groups. The level of PAB gradually decreased with growing hospital stay in fatal patients but had an increased trend in recovered patients (Supplementary Figure 1). These data suggest that the dynamic monitoring of PAB provides a potential value for mastering the process of the disease. These findings would also alert clinicians to pay special attention not only to inflammatory indexes but also to nutritional and immune status.

Some limitations in this study should be addressed. First, since the number of participants recruited in this one-center study is limited, a further design with a large multicenter cohort will provide more conclusive and valuable data. Second, some indicators, such as PAB and DD, were over the detection limit, which would lead to bias. Finally, medical history such as malnutrition and the use of steroid drugs were not included in the regression analysis and it would affect our results.

Collectively, our study provides the evidence that PAB level on admission could be used to predict in-hospital mortality in patients with COVID-19. The information provided in our study shows potential value in enriching knowledge about this critical disease, helping clinicians to identify patients with poor prognosis at an early stage before they die from COVID-19, guiding appropriate and effective management for future patients, and consequently helping to improve patients' outcomes and decrease the fatality rate.

## Data Availability Statement

All datasets presented in this study are included in the article/Supplementary Material.

## Ethics Statement

This study was reviewed and approved by the ethical committee of Tongji Hospital, Tongji Medical College, Huazhong University of Science and Technology, Wuhan, China (TJ-C20200128). The patients/participants provided their written informed consent to participate in this study.

## Author Contributions

YL, FW, and ZS conceived the research, designed the study, interpreted data, and wrote the manuscript. YL, YX, LM, and XY contributed to the acquisition of clinical data. YL, QL, GT, and HS recruited the participants, performed experiments, and analyzed data. All authors approved the final draft of the manuscript.

## Conflict of Interest

The authors declare that the research was conducted in the absence of any commercial or financial relationships that could be construed as a potential conflict of interest.

## Supplementary Material

Supplementary Figure 1 Dynamic monitoring of PAB in the representative of fatal and recovered patients. **(A)** Line diagrams showing the level of PAB in four representatives of the fatal group. **(B)** Line diagrams showing the level of PAB in four representatives of the recovered group. ^*^days from admission onset to detection. PAB, prealbumin.

Supplementary Table 1 Logistic regression analysis.

## References

[1] Holshue ML DeBolt C Lindquist S Lofy KH Wiesman J Bruce H First case of 2019 novel coronavirus in the United States. N Engl J Med. (2020) 382:929–36. 10.1056/NEJMoa2001191 32004427 PMC7092802

[2] Zhu N Zhang D Wang W Li X Yang B Song J. A novel coronavirus from patients with pneumonia in China, 2019. N Engl J Med. (2020) 382:727–33. 10.1056/NEJMoa2001017 31978945 PMC7092803

[3] Chen T Wu D Chen H Yan W Yang D Chen G. Clinical characteristics of 113 deceased patients with coronavirus disease 2019: retrospective study. BMJ. (2020) 368:m1091. 10.1136/bmj.m1091 32217556 PMC7190011

[4] Richardson S Hirsch JS Narasimhan M Crawford JM McGinn T Davidson KW. Presenting characteristics, comorbidities, and outcomes among 5700 patients hospitalized with COVID-19 in the New York City Area. JAMA. (2020) 323:2052–9. 10.1001/jama.2020.6775 32320003 PMC7177629

[5] World Health Organization Novel Coronavirus (2019-ncov) Situation Report 108. Available online at: https://www.who.int/emergencies/diseases/novel-coronavirus-2019/situation-reports/ (accessed May 8, 2020).

[6] Chen R Liang W Jiang M Guan W Zhan C Wang T. Risk factors of fatal outcome in hospitalized subjects with coronavirus disease 2019 from a nationwide analysis in China. Chest. (2020). 10.1016/j.chest.2020.04.010. [Epub ahead of print]. 32304772 PMC7158802

[7] Zhang J Wang X Jia X Li J Hu K Chen G. Risk factors for disease severity, unimprovement, and mortality in COVID-19 patients in Wuhan, China. Clin Microbiol Infect. (2020) 26:767–772. 10.1016/j.cmi.2020.04.012 32304745 PMC7159868

[8] Li X Xu S Yu M Wang K Tao Y Zhou Y Risk factors for severity and mortality in adult COVID-19 inpatients in Wuhan. J Allergy Clin Immunol. (2020). 10.1016/j.jaci.2020.04.006. [Epub ahead of print]. PMC7152876 32294485

[9] Zheng Z Peng F Xu B Zhao J Liu H Peng J Risk factors of critical & mortal COVID-19 cases: A systematic literature review and meta-analysis. J Infect. (2020). 10.1016/j.jinf.2020.04.021. [Epub ahead of print]. PMC7177098 32335169

[10] Gao L Jiang D Wen XS Cheng XC Sun M He B. Prognostic value of NT-proBNP in patients with severe COVID-19. Respir Res. (2020) 21:83. 10.1186/s12931-020-01352-w 32293449 PMC7156898

[11] Henry BM de Oliveira MHS Benoit S Plebani M Lippi G. Hematologic, biochemical and immune biomarker abnormalities associated with severe illness and mortality in coronavirus disease 2019 (COVID-19): a meta-analysis. Clin Chem Lab Med. (2020) 58:1021–8. 10.1515/cclm-2020-0369 32286245

[12] Du R-H Liang L-R Yang C-Q Wang W Cao T-Z Li M. Predictors of mortality for patients with COVID-19 pneumonia caused by sars-CoV-2: a prospective cohort study. Eur Respir J. (2020) 55:2000524. 10.1183/13993003.00524-2020 32269088 PMC7144257

[13] Tang N Li D Wang X Sun Z. Abnormal coagulation parameters are associated with poor prognosis in patients with novel coronavirus pneumonia. J Thromb Haemost. (2020) 18:844–7. 10.1111/jth.14768 32073213 PMC7166509

[14] Yang X Yang Q Wang Y Wu Y Xu J Yu Y. Thrombocytopenia and its association with mortality in patients with COVID-19. J Thromb Haemost. (2020) 18:1469–72. 10.1111/jth.14848 32302435 PMC9906135

[15] Zhang L Yan X Fan Q Liu H Liu X Liu Z. D-dimer levels on admission to predict in-hospital mortality in patients with Covid-19. J Thromb Haemost. (2020) 18:1324–29. 10.1111/jth.14859 32306492 PMC7264730

[16] Hou H Zhang B Huang H Luo Y Wu S Tang G. Using IL-2R/lymphocyte for predicting the clinical progression of patients with COVID-19. Clin Exp Immunol. (2020) 201:76–84. 10.1111/cei.13450 32365221 PMC7267633

[17] Wang F Hou H Luo Y Tang G Wu S Huang M. The laboratory tests and host immunity of COVID-19 patients with different severity of illness. JCI Insight. (2020) 5:e137799. 10.1172/jci.insight.137799 32324595 PMC7259533

[18] Liu F Li L Xu M Wu J Luo D Zhu Y. Prognostic value of interleukin-6, C-reactive protein, and procalcitonin in patients with COVID-19. J Clin Virol. (2020) 127:104370. 10.1016/j.jcv.2020.104370 32344321 PMC7194648

[19] Lagunas-Rangel FA Chávez-Valencia V. High IL-6/IFN-γ ratio could be associated with severe disease in COVID-19 patients. J Med Virol. (2020). 10.1002/jmv.25900. [Epub ahead of print]. 32297995 PMC7262117

[20] Braga M Vignali A Gianotti L Cestari A Profili M Carlo VD. Immune and nutritional effects of early enteral nutrition after major abdominal operations. Eur J Surg. (1996) 162:105–12. 8639722

[21] Moulias R Devillechabrolle A Lesourd B Proust J Marescot MR Doumerc S. Respective roles of immune and nutritional factors in the priming of the immune response in the elderly. Mech Ageing Dev. (1985) 31:123–37. 10.1016/S0047-6374(85)80023-3 3877221

[22] Loftus TJ Brown MP Slish JH Rosenthal MD. Serum Levels of Prealbumin and Albumin for Preoperative Risk Stratification. Nutr Clin Pract. (2019) 34:340–8. 10.1002/ncp.10271 30908744

[23] Gunerhan Y Koksal N Sahin UY Uzun MA Eksioglu-Demiralp E. Effect of preoperative immunonutrition and other nutrition models on cellular immune parameters. World J Gastroenterol. (2009) 15:467–72. 10.3748/wjg.15.467 19152452 PMC2653369

[24] Xu B Fan C-Y Wang A-L Zou Y-L Yu Y-H He C. Suppressed T cell-mediated immunity in patients with COVID-19: a clinical retrospective study in Wuhan, China. J Infect. (2020) 81:e51–60. 10.1016/j.jinf.2020.04.012 32315725 PMC7166040

[25] Liu Z Long W Tu M Chen S Huang Y Wang S. Lymphocyte subset (CD4+, CD8+) counts reflect the severity of infection and predict the clinical outcomes in patients with COVID-19. J Infect. (2020). 10.1016/j.jinf.2020.03.054. [Epub ahead of print]. 32283159 PMC7151318

[26] Fu L Wang B Yuan T Chen X Ao Y Fitzpatrick T. Clinical characteristics of coronavirus disease 2019. (COVID-19) in China: a systematic review and meta-analysis. J Infect. (2020) 80:656–65. 10.1016/j.jinf.2020.03.041 32283155 PMC7151416

[27] Guo W Li M Dong Y Zhou H Zhang Z Tian C Diabetes is a risk factor for the progression and prognosis of COVID-19. Diabetes Metab Res Rev. (2020) 2020:e3319 10.1002/dmrr.3319 PMC7228407 32233013

[28] DeLong ER DeLong DM Clarke-Pearson DL Comparing the areas under two or more correlated receiver operating characteristic curves: a nonparametric approach. Biometrics. (1988) 44:837–45. 10.2307/2531595 3203132

[29] Spiteri G Fielding J Diercke M Campese C Enouf V Gaymard A. First cases of coronavirus disease 2019 (COVID-19) in the WHO European Region, 24 January to 21 February 2020. Euro Surveill. (2020) 25:2000178. 10.2807/1560-7917.ES.2020.25.9.2000178 32156327 PMC7068164

[30] Yuan J Li M Lv G Lu ZK. Monitoring transmissibility and mortality of COVID-19 in Europe. Int J Infect Dis. (2020) 95:311–15. 10.1016/j.ijid.2020.03.050 32234343 PMC7102547

[31] Escalera-Antezana JP Lizon-Ferrufino NF Maldonado-Alanoca A Alarcon-De-la-Vega G Alvarado-Arnez LE Balderrama-Saavedra MA. Clinical features of the first cases and a cluster of Coronavirus Disease 2019 (COVID-19) in Bolivia imported from Italy and Spain. Travel Med Infect Dis. (2020) 2020:101653. 10.1016/j.tmaid.2020.101653 32247926 PMC7129170

[32] National Institute for H, Care Excellence in collaboration with NHSE, Improvement NHS. Managing COVID-19 symptoms (including at the end of life) in the community: summary of NICE guidelines. BMJ. (2020) 369:m1461. 10.1136/bmj.m1461 32312715

[33] Phua J Weng L Ling L Egi M Lim C-M Divatia JV. Intensive care management of coronavirus disease 2019 (COVID-19): challenges and recommendations. Lancet Resp Med. (2020). 8:506–17. 10.1016/S2213-2600(20)30161-2 32272080 PMC7198848

[34] Wu C Chen X Cai Y Xia J Zhou X Xu S. Risk factors associated with acute respiratory distress syndrome and death in patients with coronavirus disease 2019 pneumonia in Wuhan, China. JAMA Intern Med. (2020) e200994. 10.1001/jamainternmed.2020.0994. [Epub ahead of print]. 32167524 PMC7070509

[35] Mkhize BT Mabaso MHL Madurai S Mkhize-Kwitshana ZL. The investigation of the use of prealbumin as a tool for nutritional assessment in adults coinfected with HIV and intestinal helminth parasites in kwazulu-natal, South Africa. Biomed Res Int. (2018) 2018:7805857. 10.1155/2018/7805857 30065944 PMC6051331

[36] Sharma A Giraddi G Krishnan G Shahi AK. Efficacy of serum prealbumin and crp levels as monitoring tools for patients with fascial space infections of odontogenic origin: a clinicobiochemical study. J Maxillofac Oral Surg. (2014) 13:1–9. 10.1007/s12663-012-0376-4 24644389 PMC3955473

[37] Bateman RM Sharpe MD Jagger JE Ellis CG Solé-Violán J López-Rodríguez M 36th international symposium on intensive care and emergency medicine: Brussels, Belgium. Crit Care. (2016) 20(Suppl 2):94 10.1186/s13054-016-1208-6 27885969 PMC5493079

[38] Hatzistilianou M. Diagnostic and prognostic role of procalcitonin in infections. ScientificWorldJournal. (2010) 10:1941–6. 10.1100/tsw.2010.181 20890583 PMC5763968

[39] Aoshi T Koyama S Kobiyama K Akira S Ishii KJ. Innate and adaptive immune responses to viral infection and vaccination. Curr Opin Virol. (2011) 1:226–32. 10.1016/j.coviro.2011.07.002 22440781

[40] Newton AH Cardani A Braciale TJ. The host immune response in respiratory virus infection: balancing virus clearance and immunopathology. Semin Immunopathol. (2016) 38:471–82. 10.1007/s00281-016-0558-0 26965109 PMC4896975

[41] Luo Y Xie Y Zhang W Lin Q Tang G Wu S. Combination of lymphocyte number and function in evaluating host immunity. Aging (Albany NY). (2019) 11:12685–707. 10.18632/aging.102595 31857499 PMC6949078

[42] Holly MK Diaz K Smith JG. Defensins in Viral Infection and Pathogenesis. Annu Rev Virol. (2017) 4:369–91. 10.1146/annurev-virology-101416-041734 28715972

[43] Hanada S Pirzadeh M Carver KY Deng JC. Respiratory viral infection-induced microbiome alterations and secondary bacterial pneumonia. Front Immunol. (2018) 9:2640. 10.3389/fimmu.2018.02640 30505304 PMC6250824

[44] Guan WJ Ni ZY Hu Y Liang WH Ou CQ He JX Clinical Characteristics of Coronavirus Disease 2019 in China. N Engl J Med. (2020) 382:1708–20. 10.1056/NEJMoa2002032 32109013 PMC7092819

[45] Zhang J Yu M Tong S Liu LY Tang LV. Predictive factors for disease progression in hospitalized patients with coronavirus disease 2019 in Wuhan, China. J Clin Virol. (2020) 127:104392. 10.1016/j.jcv.2020.104392 32361327 PMC7187844

