# Supplementary figures and images for: Prealbumin as a Predictor of Prognosis in Patients With Coronavirus Disease 2019
Source: Front Med (Lausanne). 2020 Jun 26;7:374. doi: 10.3389/fmed.2020.00374 (PMC7333015; doi:10.3389/fmed.2020.00374)

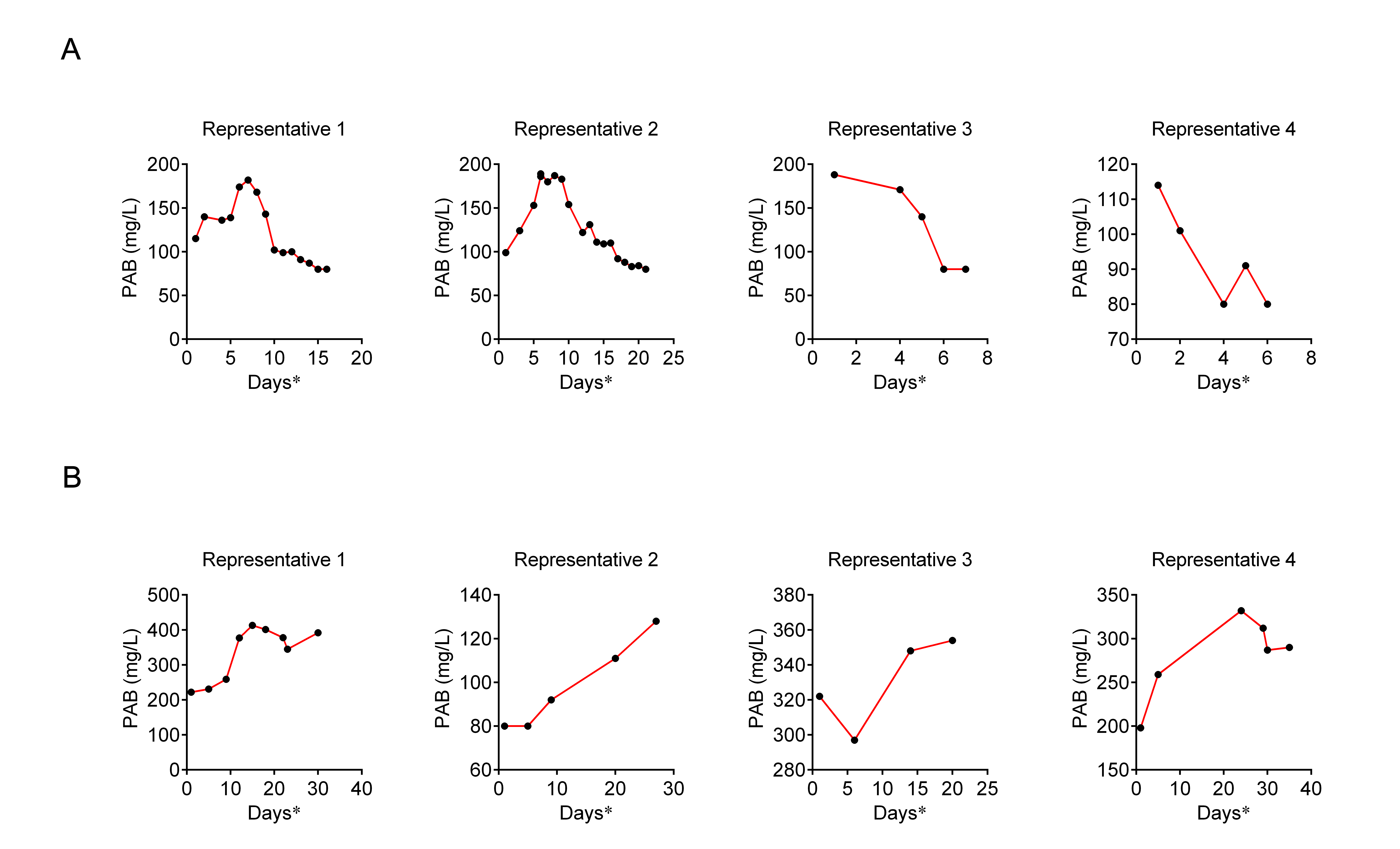

Supplement: Supplementary Figure 1 — Dynamic monitoring of PAB in the representative of fatal and recovered patients. (A) Line diagrams showing the level of PAB in four representatives of the fatal group. (B) Line diagrams showing the level of PAB in four representatives of the recovered group. *days from admission onset to detection. PAB, prealbumin. [file Image_1.TIF]
