# Supplementary material for: Prealbumin as a Predictor of Prognosis in Patients With Coronavirus Disease 2019
Source: Front Med (Lausanne). 2020 Jun 26;7:374. doi: 10.3389/fmed.2020.00374 (PMC7333015; doi:10.3389/fmed.2020.00374)
Supplement: Supplementary Table 1 — Logistic regression analysis. [file Table_1.DOCX]

Supplementary Table 1. Logistic regression analysis.

|  | | | | | | | |
| --- | --- | --- | --- | --- | --- | --- | --- |
|  | | B | S.E | Wals | df | Sig. | Exp(B) |
|  | LYM# | -.908 | .344 | 6.980 | 1 | .008 | .403 |
|  | NEU# | .067 | .030 | 4.810 | 1 | .028 | 1.069 |
|  | PAB | -.016 | .003 | 38.486 | 1 | .000 | .984 |
|  | PCT | .060 | .033 | 3.329 | 1 | .048 | 1.062 |
|  | hsCRP | .005 | .002 | 4.854 | 1 | .028 | 1.005 |
|  | PT | .154 | .080 | 3.692 | 1 | .045 | 1.167 |
|  | LDH | .003 | .001 | 15.796 | 1 | .000 | 1.003 |
|  | CR | .002 | .001 | 2.998 | 1 | .043 | 1.002 |
|  | hs-cTnI | .001 | .000 | 2.042 | 1 | .049 | 1.001 |
|  | constant | -3.036 | 1.410 | 4.633 | 1 | .031 | .048 |
|  | | | | | | | |
